# COVID-19 in congenital heart disease patients: what did we learn?!

**DOI:** 10.3389/fcvm.2023.1235165

**Published:** 2023-08-31

**Authors:** Rana Zareef, Elio Salameh, Rawan Hammoud, Theresia Tannouri, Fadi Bitar, Mariam Arabi

**Affiliations:** ^1^ Pediatric and Adolescent Medicine Department, American University of Beirut Medical Center, Beirut, Lebanon; ^2^ Faculty of Medicine, American University of Beirut Medical Center, Beirut, Lebanon; ^3^ Pediatric and Adolescent Medicine Department, Division of Pediatric Cardiology, American University of Beirut Medical Center, Beirut, Lebanon

**Keywords:** COVID-19, SARS-CoV-2, coronavirus, congenital heart disease, congenital cardiac abnormalities

## Abstract

**Aim:**

COVID-19 pandemic has spread widely at unpreceded pace. Cardiovascular comorbidities are significantly correlated with severe and critical illness. Nevertheless, studies examining the impact of congenital heart disease on COVID-19 severity are scarce and restricted to hospitalized patients. This study aims to explore the course of COVID-19 illness, severity and complications in patients with concomitant congenital heart disease.

**Methodology:**

This study is a cross sectional survey that includes patients with congenital heart disease who are registered at the Children Heart Center at the American University of Beirut Medical Center. The survey was conducted in the pediatric cardiology outpatient clinics or remotely via phone calls.

**Results:**

A total of 238 patients participated in the study, of which 47.9% had suspected or confirmed diagnosis of SARS-CoV-2 infection. The majority of patients had mild illness. The symptoms ranged between rhinorrhea (15.6%), cough (15.6%), low-grade fever (11.2%), anosmia (2.7%), ageusia (2.5%), headache (9.8%), high-grade fever (8.5%), gastrointestinal symptoms (7.6%), lethargy (6.3%), muscle aches (5.6%), difficulty breathing (5.4%), joint pain (4.7%), and chills (4.7%). 20% of the surveyed patients required treatment at home. Hospitalization and oxygen therapy was required in 3.5% of cases, while only 1.5% demanded intensive care admission. Only one fatality was encountered.

**Conclusion:**

COVID-19 infection in pateints with congenital heart disease exhibits a mild to moderate course, and doesn't necessarily increase risk of complicated disease. No correlation could be found between specific congenital heart lesion and disease severity.

## Introduction

In December 2019, an outbreak of atypical pneumonia was recorded in Wuhan, China (1). Shortly thereafter, events escalated rapidly and led to the emergence of the global COVID-19 pandemic (1). The novel virus SARS-CoV-2 was identified as the causative agent (2). It is a single stranded RNA virus and a member of the coronaviridae family (2). COVID-19 has engendered an array of symptoms and has fostered severe complicated diseases with muti-system involvement in some instances (3). Consequently, it has precipitated considerable worldwide mortality and morbidity in a short period (1). With the extensive proliferation of the virus, children have also been struck. Fortunately, evidence in the literature that delineates the course of COVID-19 disease in children is reassuring (4). They usually procure mild forms of illness, and most are even asymptomatic (5). On the contrary, studies showed that patients with co-existing cardiovascular conditions are at higher risk of experiencing severe and complicated illness (6, 7). In addition, severe form of COVID-19 can hasten cardiovascular insults such as heart failure, arrythmias, ventricular depression, myocarditis, and critical thromboembolic events (8). Even in children, COVID-19 has provoked myocardial injury, arrhythmias, and valvular abnormalities (9). Therefore, discussion should be raised towards a critical population: those with congenital heart disease.

Congenital heart disease is the most common congenital anomaly, accounting for around 1% of all live births (10). It exhibits a wide variety of symptoms, from those who are completely asymptomatic to those with profound disease that limits the quality of life (10). Indeed, the prognosis of children with congenital heart disease has improved significantly over the years with the improvement and advances in medical and surgical techniques (11). However, this population remains at a particular risk of acquiring infectious organisms that might influence the respiratory tract (12). Hence, concerns are raised regarding COVID-19 infection in patients with congenital heart lesions. Despite the extraordinary speed in uncovering scientific facts related to COVID-19, a gap in the literature exists as regards to the disease severity and prognosis in patients with congenital heart disease. Studies that tackle this population have shed the light on those who required hospitalization. Nevertheless, a comprehensive investigation of the disease course, severity, prognosis, and risk factors in this specific category of patients is missing. This study aims to investigate the course, severity, complications, and outcome of COVID-19 illness in patients with congenital heart disease.

## Methodology

To evaluate the disease severity and course of illness of COVID-19 in patients with congenital heart disease, we conducted a single-center cross-sectional survey at the Children Heart Center (CHC) at the American University of Beirut Medical Center (AUBMC). The study was conducted between January 2022 and July 2022. After obtaining the approval of the institutional review board at the AUBMC, patients with CHD who are registered at CHC, their parents, or legal guardians were randomly contacted and asked to participate in the survey. Due to COVID-19 pandemic, the study was conducted physically through filling surveys in the pediatric cardiology outpatient clinics or remotely through phone calls to minimize direct contact. Patients were randomly approached either when they arrived to the outpatient pediatric cardiology clinic for a regular visit or via phone calls. Patients, parents, or legal guardians, if agreed to participate in the study, were asked to fill out a questionnaire related to COVID-19 infection status, patient's baseline functional status, concomitant chronic illness, chronic medication use, symptoms of COVID-19 disease, complications, need for hospitalization, and need for intensive care admission. The study included both children and adults with CHD. The diagnosis of SARS-CoV-2 infection was defined by positive reverse transcription polymerase chain reaction (RT-PCR), antigen test, chest imaging suggestive of COVID-19, or clinically. In addition, some patients were clinically suspected of having COVID-19 without definitive diagnosis. Data regarding COVID-19 symptoms, illness severity, duration of symptoms, need for hospitalization, need for intensive care admission, oxygen requirement, treatment strategy, and presence of possible complications were all collected and analyzed.

### Statistical analysis

All analysis was conducted and represented using Microsoft Excel and SPSS 29.0.0.0. Discrete variables were represented as absolute numbers or percentages of the total. Mean and standard deviations were calculated and included for all normally distributed continuous variables and were reported as mean ± standard error of the mean.

## Results

### Demographics

**Figure 1:**
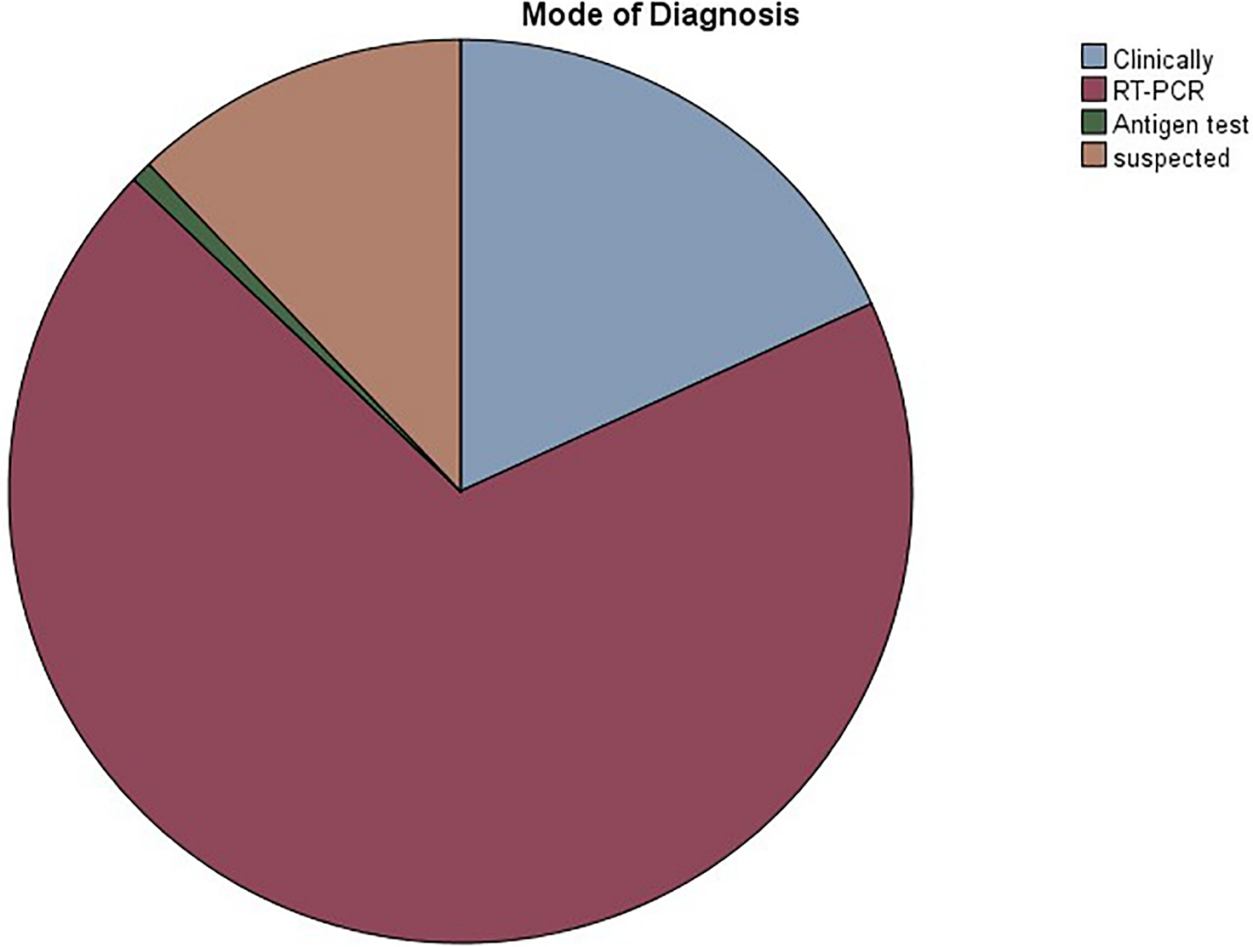
Pie chart showing the distribution of COVID-19 mode of diagnosis in the study participants.

**Figure 2:**
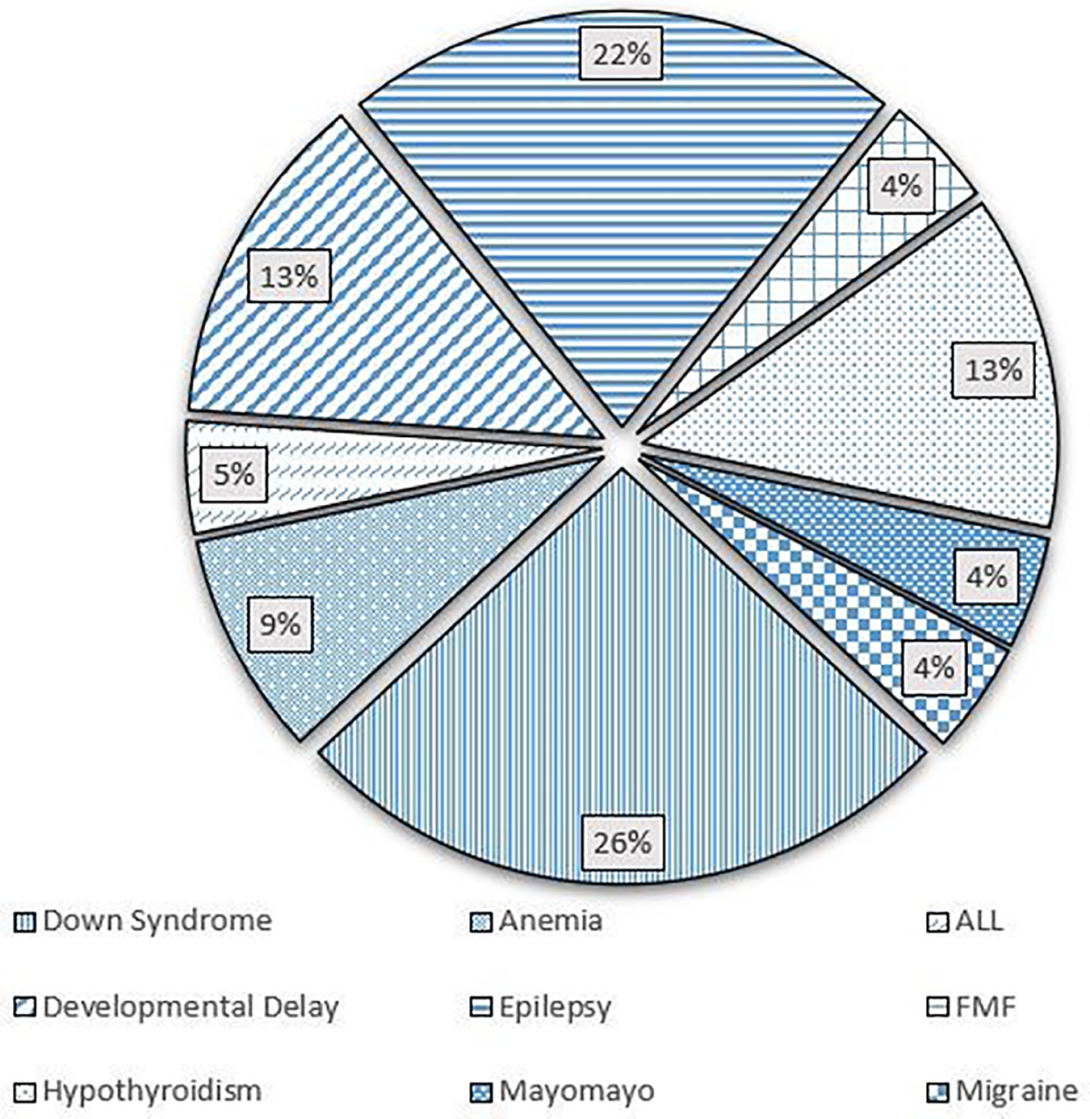
Pie chart showing the distribution of chronic disease in the study participants.

The study included 238 patients, with mean age of 8.02 ± 7.45 years. In our population, 50.8% were males, while 49.2% were females. A total of 124 (52.1%) patients had a confirmed or suspected diagnosis of SARS-CoV-2 infection. A summary of the demographic characteristics of the patients is included in Table 1. Of the patients who had confirmed or suspected diagnosis of SARS-CoV-2 infection, five didn't share their age at infection. The mean age of the remaining 119 patients was 9.43 ± 7.98 years. The youngest patient was a 2 months old, while the eldest was 43. Five patients didn't specify a gender, while females accounted for 49.6% of the remaining patients. 118 (95.2%) patients reported a normal and active pre-infection status. The majority (68.9%) were diagnosed via a positive RT-PCR, while 18.2% reported a clinical diagnosis, 0.8% were diagnosed by antigen test, and 12.1% were only suspected of having the infection, as seen in Figure 1. Interestingly, 60.7% of patients in the study had one or several family members infected with the virus, a percentage that increases to 96.7% when limited to those diagnosed with SARS-CoV-2 infecton. Out of the 124 who contracted the virus, 24 (19.4%) had at least one concomitant chronic disease, and 52 (43%) patients were on chronic medications. Figure 2 displays the distribution of chronic diseases. Of those maintained on cardiovascular medications, 20 patients were on aspirin, seven were on furosemide, nine were maintained on oral anticoagulation, eight on beta blockers (one Carvedilol, three Atenolol, and four propranolol), and 14 maintained on ACE inhibitor (nine on enalapril and five on captopril). The rest of the medications were prescribed for non-cardiovascular causes. In addition, Table 2 displays the distribution of patients according to the congenital heart anomaly. Fifteen patients (12.1%) had an unspecified CHD. Tetralogy of Fallot was the most common congenital anomaly (13.7%), followed by the presence of single or multiple VSDs (9.7%).

**Table 1:** The demographic characteristics of the patients is included in the study.

Characteristic	Frequency (%)	Total number of responses
Mean age, whole sample	8.02 ± 7.45 years	175
Gender, whole sample:		173
Male	88 (50.8)	
Female	85 (49.2)	
COVID-19 infection:		238
Confirmed/suspected	124 (52.1)	
Non-infected	114 (47.1)	
Pre-infection status:		124
Active	118 (95.2)	
Inactive	6 (4.8)	
Infected family members:		
Of all patients in the study	60.7%	
Of infected patients	96.7%	
Mean age, of infected patients	9.43 ± 7.98 years	
Gender, infected patients:		119
Male	60 (50.4)	
Female	59 (49.6)	
Chronic disease in infected patients:		124
Present	24 (19.4)	
Absent	100 (80.6)	
Chronic medication use in infected patients:		124
Present	52 (41.9)	
Absent	72 (58.1)	

**Table 2:** Distribution according to the congenital heart anomaly.

Type of congenital anomaly	Frequency (%)
CHD, unspecified	15 (12.1)
ASD	12 (9.7)
AVC	12 (9.7)
Bicuspid AV	5 (4)
Bicuspid AV, AR, dilated AA	1 (0.8)
CoA	5 (4)
Dilated AA	1 (0.8)
Dilated cardiomyopathy	1 (0.8)
DORV	1 (0.8)
Ebstein's anomaly	2 (1.6)
HOCM	2 (1.6)
Interrupted aortic arc type B with large VSD	1 (0.8)
Mitral atresia	1 (0.8)
MVR	2 (1.6)
MVP	3 (2.4)
PA	3 (2.4)
PS	9 (7.3)
SV	4 (3.2)
Subaortic membrane	1 (0.8)
TA	6 (4.8)
TA + VSD + PS + hypoplastic RV	1 (0.8)
TGA	6 (4.8)
TOF	17 (13.7)
Single or multiple VSDs	12 (9.7)
VSD + ASD	1 (0.8)
**Total**	**124**

ASD, atrial septal defect; AVC, Atrioventricular canal; AR, aortic regurgitation; AA, ascending aorta; CoA, Coarctation of the aorta; DORV, double outlet right ventricle; HOCM, hypertrophic obstructive cardiomyopathy; VSD, ventricular septal defect; MVR, Mitral valve regurgitation; MVP, Mitral valve prolapse; PA, pulmonary atresia; PS, pulmonary stenosis; SV, single ventricle; TA, tricuspid atresia; RV, right ventricle; TGA, transposition of great arteries; TOF, tetralogy of Fallot.

### Disease course and severity

**Figure 3:**
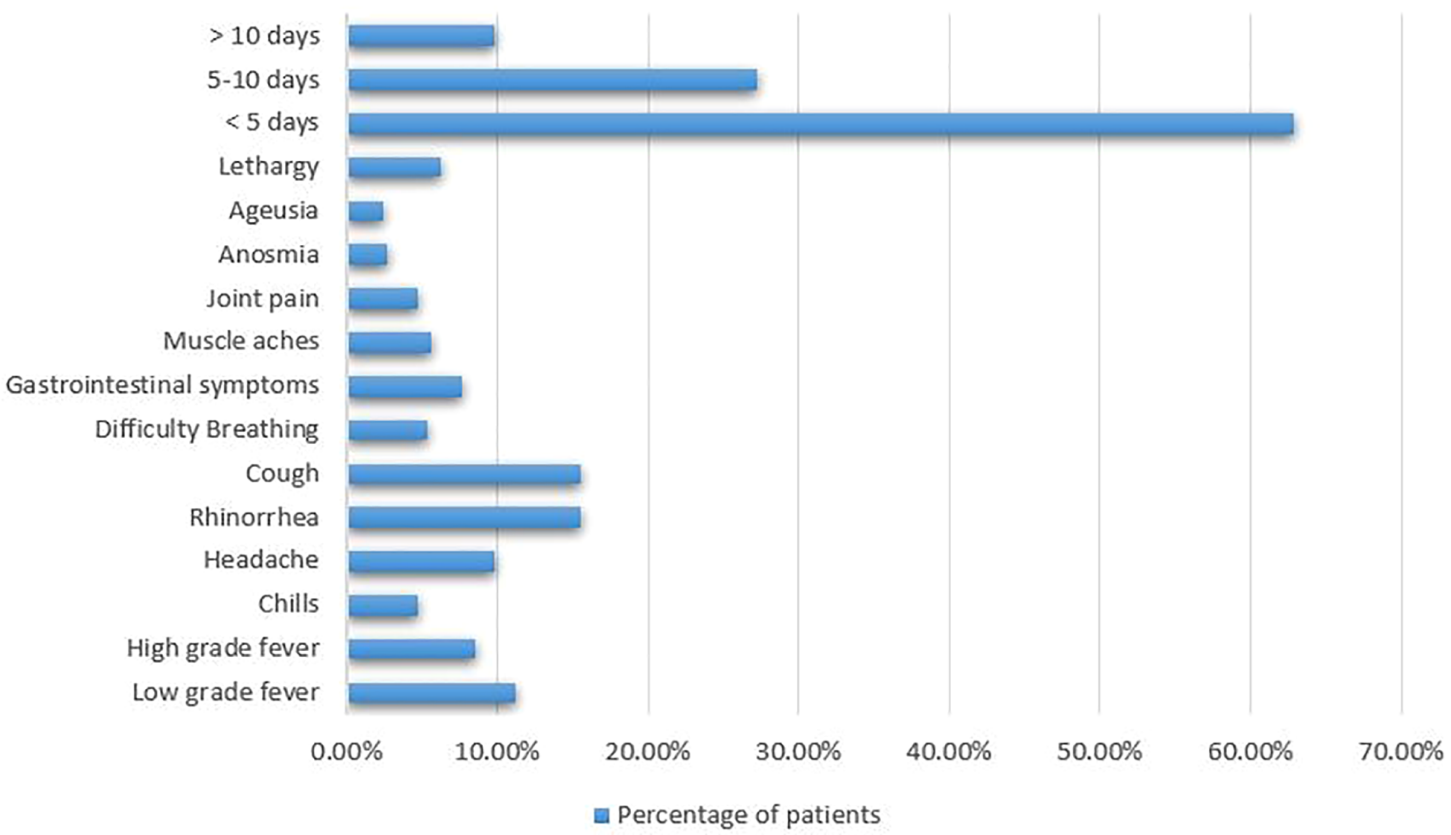
Histogram showing the percentage of patients experiencing each symptom reported.

Symptoms of COVID-19 disease ranged widely from rhinorrhea (15.6%), cough (15.6%), and low-grade fever (11.2%) to anosmia (2.7%) and ageusia (2.5%). Many patients also experienced headache (9.8%), high-grade fever (8.5%), gastrointestinal symptoms (7.6%), lethargy (6.3%), muscle aches (5.6%), difficulty breathing (5.4%), joint pain (4.7%), and chills (4.7%). Most of these patients (62.9%) experienced their symptoms for a duration less than 5 days, while 27.3% and 9.8% had their symptoms for 5-10 days and for more than 10 days, respectively, as can be seen in Figure 3.

20% of patients received treatment at home, while 80% did not. Also, 29.2% reported that they have sought medical attention. On the other hand, 96.2% of infected patients did not require hospitalization nor oxygen support, while a minority (3.8%) did require them both. In addition, only 1.5% of patients required intensive care admission, whereas the vast majority (98.5%) did not.

The majority (93.9%) of CHD patients did not report any complications after their infection. The most common complication was pneumonia, reported in 75% of those with complications (6.1% of the total), while pericarditis (12.5%) and spells requiring urgent surgery (12.5%) constituted the rest. Table 3 represents the clinical characteristics of patients who reported complications secondary to COVID-19 infection.

**Table 3:** Clinical characteristics of CHD patients who developed complications following COVID-19 infection.

Age group	Complication	Comorbidities	Hospital admission	ICU admission	Heart lesion
Young adult	Pneumonia	Asthma	No	No	CHD, unspecified
Adolescent	Pneumonia	None	Yes	Yes	CHD, unspecified
Adolescent	Pneumonia	None	No	No	VSD
Child	Pneumonia	Asthma	No	No	TOF
NA	Pneumonia	None	No	No	CHD, Unspecified
Adult	Pericarditis	None	Yes	No	MVR
Child	Pneumonia	Developmental delay + Epilepsy	No	No	MA
Child	Infection has precipitated spells, and required urgent surgical intervention.	Down syndrome	Yes	Yes	TA/VSD/PS/hypoplastic RV

Only one case of death was reported. A 17 months old male with down syndrome and severe form of TOF, and who required previous BT-shunt insertion during the neonatal period. He had a history of recurrent hospitalization due to frequent upper respiratory tract infections. He was maintained on furesoamide and captopril at home, and was planned for cardiac catheterization followed by corrective surgery. He contracted SARS-CoV-2 virus and required hospitalization for monitoring and supportive care. The patient was hospitalized in another hospital where the death occurred; therefore, extensive medical information regarding their hospital stay and complications couldn't be obtained.

## Discussion

COVID-19 pandemic has evolved rapidly and widely impacted the various populations. This study highlights the medical impact of COVID-19 on a small yet special subset of patients: those with congenital heart disease. The current study reports the course of COVID-19 disease in 124 patients with various cyanotic and acyanotic congenital heart anomalies. Our population age was skewed to the lower side, with average age of 9.43 ± 7.98 years. The majority were diagnosed via RT-PCR, while a small subset (12.1%) was suspected to have contracted the virus without definitive diagnosis. Overall, the disease followed a mild path with a short duration, while only 6% of patients developed complications. No fatalities were encountered. Low grade fever, rhinorrhea, and cough were the most reported symptoms. Low percentage of patients experienced anosmia, ageusia, arthralgias and myalgias, possibly because it is difficult to report such symptoms in young children.

Among those who developed complications, we couldn't identify a specific heart lesion predisposing to severe disease. This is attributed to the low number of complications encountered. Interestingly, a good proportion of patients received medical treatment at home (20%), even with the absence of complications. Besides, 70.8% didn't seek medical attention, or contact their primary care physician, possibly due to the asymptomatic to mild disease presentation. Our study suggests that congenital heart lesion doesn't necessarily increase the risk of developing severe and critical illness. Another study conducted by Langes et al. (13) on adult patients with CHD showed that among those diagnosed with SARS-CoV-2 infection, 50% were asymptomatic and approximately 40% were unaware of their infectious status. In our population, only 3.8% required hospitalization and 1.5% were admitted to the intensive care unit. Additionly, complications and severe illness are suggested to be associated with poor baseline CHD status and function. Our population exhibited good and active pre-infection status. The disease complications and mortality increase parallel to the patient's age and morbidities. However, most of the included patients in our study are children with comorbidities limited to CHD, which might have positively influenced the results. Only 19.4% suffer a concomitant chronic illness. A study by Sabatino and colleagues including patients from the Italian Congenital Heart Disease Units goes in line with our results. Investigators concluded that the disease course of COVID-19 was not more severe than the general population, as was shown in other cardiovascular diseases. Cardiovascular complications were mainly observed in the adults with CHD group (14). In addition, the mortality rate for COVID-19 in CHD patients was 2.3%, similar to that of the general population, which is 2.2% (15). Also, the structural or anatomical defects observed in CHD did not correlate with a higher risk of COVID-19 severity or fatality rate, suggesting that it was other risk factors responsible for the increased morbidity including diabetes, renal insufficiency, age, sex, etc.

Of the patients who experienced complications secondary to the infection, 6 out of 8 patients developed pneumonia and required treatment with antibiotics. One patient had severe pneumonia requiring prolonged hospitalization, oxygen therapy and 4-day ICU admission. Another young child had precipitation of cyanotic spells that was resistant to medical therapy. The decision was made to intervene surgically. This patient had poor baseline functional status and their medical history is complicated by the diagnosis of down syndrome. We failed to draw an association between the incidence of complications following COVID-19 infection and the degree of CHD severity, due to the small number of encountered complications. In fact, it is suggested that patients with depressed left ventricular function, critical pulmonary hypertension, significant ischemia, unrepaired heart lesion, and associated genetic syndrome are at increased risk of experiencing severe and critical illness. A case series of nine pediatric patients with COVID-19 and concomitant CHD at the Children Medical Center Hospital in 2020 (16) showed that a mild course of COVID-19 is in fact the general case of the pediatric population, even those with a mild CHD. Nevertheless, the symptoms as well as the clinical and prognostic outcomes worsen in children with severe forms of CHD, namely aortic valve stenosis and hypoplastic left heart syndrome, as seen in two different patients in this study who died due to worsening hypoxemia and respiratory distress. The deceased patients had worse arterial blood gas outcomes, higher CRP levels indicative of severe inflammation, as well as a higher partial thromboplastin time indicative of a concomitant coagulopathy (16). Another study conducted by Schwerzmann et al. (17) included 105 CHD patients from different European health centers with subsequent COVID-19 infection. It showed that the severity of the clinical outcomes and prognosis depended on the risk factors of the general population which include obesity, age, and other comorbidities. It added that the severity of the cardiac defect also correlated with the outcomes, in such a way that cyanotic heart lesions were more likely to result in a severe COVID-19 course.

Most of the previous studies focused on analyzing COVID-19 disease in hospitalized CHD patients. This would result in higher rates of reported complications, more severe presentation, increased need for oxygen therapy, higher intensive care admissions, and higher mortality rate. In addition, the published data consists of case series, retrospective studies, or review of the literature, which hold a high rate of innate bias. This has created wide controversy regarding the fragility of patients with CHD in COVID-19 disease. This study sheds the light on CHD patients who have contracted COVID-19 disease without necessarily requiring medical attention. A few papers suggested that CHD patients had a higher risk of having moderate to severe COVID-19 infections. One paper showed that hospitalization of CHD patients following COVID-19 infection results in more severe outcomes, especially in children, who recorded a higher mortality rate than usual (18). Furthermore, a longer hospital length of stay, higher costs, and higher morbidity were also the case of both adult and pediatric CHD patients diagnosed with COVID-19 (19). In addition, 94 CHD patients with COVID-19 from 24 Indian pediatric health centers were included in a study organized by Sachdeva et al. (20). The in-hospital mortality rate was significantly higher in COVID-19 positive cases than in negative ones, suggesting that this viral infection could put additional risk on the CHD patients who would then require further attention. In short, the available literature is highly controversial, raising doubts on the role of CHD in predicting COVID-19 disease severity. More studies are definetly needed to be able to confidently propose or reject a correlation.

### Limitations

Our study exhbits several limitations. First, the cross-sectional nature of the study provides inherent bias. Second, the study was conducted in one center and included a relatively small number of patients. Besides, children compromised the majority of the included population, therefore masking a critical risk factor: age and other comorbidities that increase in prevalence with advancement in age. Adult patients with CHD, in addition to their age, have higher chance of possessing additional comorbidities and receiving more medications. In addition, we failed to reach correlations or to dentify risk factors for complications and severe disease, as the number of such encountered outcomes was very small. Therefore, statistical analysis and correlation testing were not performed. Besides, including the vaccination status and the area of residence would have provided further essential information and guided the analysis. Moreover, not all patients were diagnosed via RT-PCR or antigen testing. A percentage of our population were diagnosed clinically or were just suspected of contracting the virus. Last but not least, the study lacks the advantage of long-term follow up to detect further outcomes especially in patients who required medical treatment, and has some missing data attributed to the cross-sectional survey nature of the study.

## Conclusion

In conclusion, our study suggests that CHD is not necessarily a risk factor of severe COVID-19. It doesn't increase the risk of experiencing complications following infection with COVID-19 when compared to the general population. In fact, severity of symptoms, duration of illness, rates of complications and hospitalization doesn't suggest a worse disease outcome when compared with the available data for the general population. Nevertheless, our study is limited by the small number of patients, and the predominantly healthy and functional baseline status of our population. Even though these results are reassuring for the CHD population, attention must still be given to the infected patients, especially those with cyanotic lesions and concomitant comorbidities, such as obesity and hypertension. Further studies with wider age range and larger sample size are needed to clearly investigate the risk factors for severe disease among this population.

## Data Availability

The original contributions presented in the study are included in the article/Supplementary Material, further inquiries can be directed to the corresponding author.
